# Grey Matter Leonardo da Vinci: a genius driven to distraction

**DOI:** 10.1093/brain/awz131

**Published:** 2019-05-28

**Authors:** Marco Catani, Paolo Mazzarello

**Affiliations:** 1 NatBrainLab, Department of Neuroimaging, Institute of Psychiatry, Psychology and Neuroscience, King's College London, De Crespigny Park, London, UK; 2 NatBrainLab, Department of Forensic and Neurodevelopmental Sciences, Institute of Psychiatry, Psychology and Neuroscience, King's College London, De Crespigny Park, London, UK; 3 Department of Brain and Behavioral Sciences and University Museum System, University of Pavia, Pavia, Italy

## The paradox of Leonardo da Vinci

Five hundred years have passed since the death of Leonardo da Vinci, and much has been written about him. Leonardo the artist, the scientist, the architect, the inventor, whose genius has been perceived as the allure of an unfathomable riddle. But some of the words written about Leonardo after he died at Clos-Lucé in France on 2 May 1519, hint at a very different man to the one many of us presume to know. According to his first biographer Giorgio Vasari, Leonardo died lamenting ‘that he had offended God and mankind in not having worked at his art as he should have done’ (Vasari, 1996; Nicholl, 2004; Vecce, 2006).

The story of Da Vinci is one of a paradox—a great mind that has compassed the wonders of anatomy, natural philosophy and art, but also failed to complete so many projects (Freud, 1922; Kemp, 2006). The excessive time dedicated to idea planning and the lack of perseverance seems to have been particularly detrimental to finalize tasks that at first had attracted his enthusiasm. Leonardo’s chronic struggle to distill his extraordinary creativity into concrete results and deliver on commitments was proverbial in his lifetime and present since early childhood:

‘in learning and in the rudiments of letters he would have made great proficiency, if he had not been so variable and unstable, for he set himself to learn many things, and then, after having begun them, abandoned them.’ (Vasari, 1996).

His difficulties with focusing became even more evident later in adolescence, when he moved from the small village of Vinci to Florence in the workshop of Andrea Verrocchio. Verrocchio, a true Renaissance man, shared Leonardo’s wide breadth of interests and eclectic talent. But Leonardo lacked his master’s rapid power of execution and organizational skills. Leonardo’s first important commissioned works, some obtained through his father’s connections, were prepared at length but quickly abandoned. Other programmed works were never started. Leonardo's struggle to work independently as an artist might also explain his unduly prolonged stay in the Verrocchio workshop lasting until the age of 26 when he probably managed to set up his own independent studio in Florence. On 10 January 1478 he received his first recorded commission as an independent painter, a large altarpiece to hang in the Chapel of San Bernardo. For this prestigious commission he obtained a cash advance of 25 florins, but he never delivered the work (Nicholl, 2004). Probably, given his unreliability in finishing the commissioned projects, he did not obtain much success as an independent painter and, unlike other artists of the Verrocchio workshop who were transferred to work in papal Rome, he was sent by Lorenzo de’ Medici to Milan as a musician (Kemp, 2006). We do not know in what state of mind Leonardo left Florence but it is possible that he felt ‘a sense of failure and frustration—his paintings unfinished, his lifestyle controversial, his reputation a mix of brilliance and difficulty’ (Nicholl, 2004). For comparison, by the same age, Raphael had already realized more than 80 paintings, including large frescos in the Vatican.

At the court of Ludovico il Moro, the future Duke of Milan, he astounded his patrons with the most ambitious ideas and projects, but failed to gain their trust in his ability to deliver on time. Even when Leonardo was finally commissioned with the important project of building a bronze statue of Ludovico’s father, the future Duke asked his allied Lorenzo il Magnifico if he could indicate a more apt Florentine artist for the project because he ‘doubted Leonardo’s capabilities to bring it to completion’ (Vecce, 2006).

**Figure 1:**
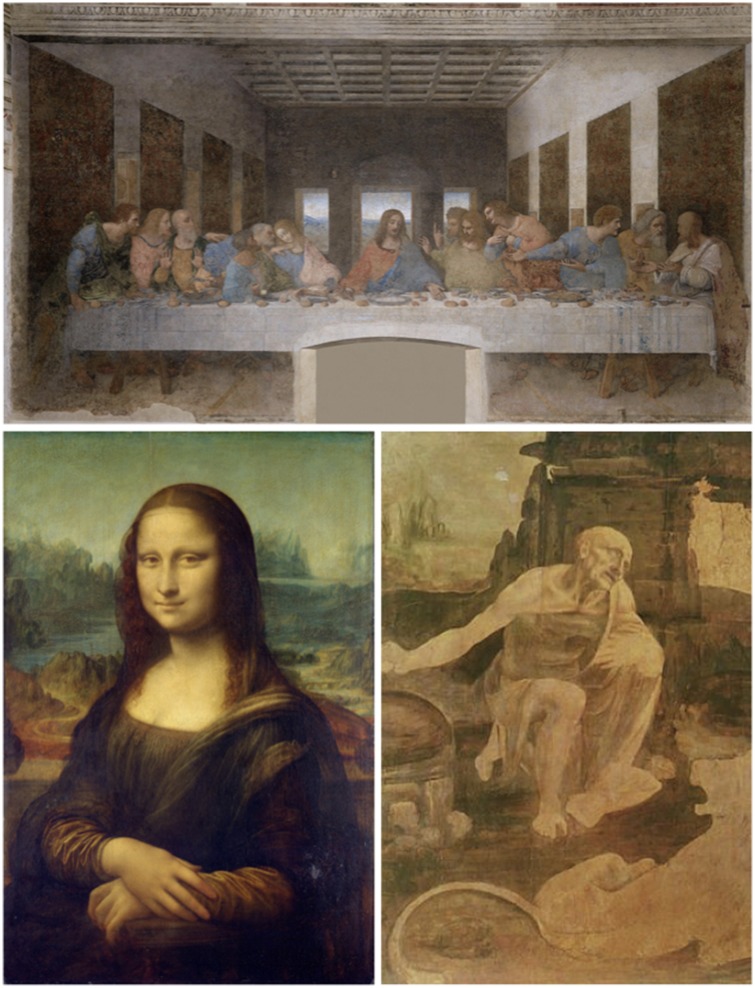
**Three of Leonardo’s masterpieces.** *Top*: The *Last supper* was completed by Leonardo in 3 years but the use of an incorrect fresco technique led to the rapid deterioration of the work. *Bottom left*: Leonardo worked intermittently on *Mona Lisa* for nearly 16 years. *Bottom right*: The unfinished painting of *Saint Jerome in the Wilderness*.

The novelist Matteo Bandello, a contemporary who observed Leonardo working on the *Last Supper* (Fig. 1), clearly identified his fickleness of temperament and chaotic organizational skills:

‘I have also seen him, as the caprice or whim took him, set out at midday, […] from the Corte Vecchio, where he was at work on the clay model of the great horse, and go straight to the Grazie and there mount on the scaffolding and take up his brush and give one or two touches to one of the figures and suddenly give up and go away again’ (Nicholl, 2004; Vecce, 2006).

Leonardo was capable of sustained contemplation or studying, but this was often at the expense of losing track of the overall progression of the project, a relentless procrastination. His unreliability was so well known that the Duke of Milan wished to have Leonardo sign a contract obliging him to finish a work ‘within the stipulated period’ (Kemp, 2006). When the Duke capitulated in 1499 and parted ways with da Vinci after almost 20 years of service, Leonardo admitted in his diary that ‘none of his projects had been finished for him’ (Vecce, 2006).

Perhaps the most disruptive side of his mind was a voracious curiosity, which both propelled his creativity and distracted him from keeping a steady path to completion. Conscious of his limits, Leonardo tried to work around them, often with unfortunate consequences. His reluctance to work on fresco painting, for example, which requires a quick execution before the plaster dries, led him to risky experiments in seeking out new oil pigments and varnish techniques that endangered the *Last Supper* and eventually destroyed the *Battaglia of Anghiari* in Florence. Such was Leonardo’s capriciousness that other artists were often called to work on paintings first commissioned to him.

Let down by his own inventiveness, Leonardo tried to team up with others who could assist him. In the winter of 1510–11 he worked with Marcantonio Della Torre, professor at the University of Pavia, to create a treatise on anatomy. Together they studied the human body and performed dissections that Leonardo beautifully depicted. This was the only period in his anatomical career during which Leonardo ‘was able to attain a balance between detail and coverage’. It was as ‘if the professional anatomist standing at his shoulder was able to save Leonardo from his habit of going ever further into the details of a physical scenario’ (Clayton and Philo, 2012). But in a matter of months, Della Torre died of plague. Alone, Leonardo never managed to organize his large number of anatomical drawings into coherent material for publication. In his notebooks he dishearteningly annotated: ‘It is easier to resist at the beginning than at the end’.

Leonardo used his wit to mask his shortcomings and talk his way out of the trouble or embarrassment caused by his behaviour. While working on the *Last Supper*, for example, he was subjected to the continuous nagging from the superintending prior of Santa Maria delle Grazie who ultimately asked the Duke of Milan for intervention. Summoned by the Duke, Leonardo quickly justified his delay with the difficulty of finding the models of the last two characters, Jesus and Judas. For Judas, he explained, he had searched in vain through the jails of Milan for the perfect looking scoundrel. None could be found and he conceded that in the end, if he could not find a better model for the cruel apostle who betrayed our Lord, he would have to use the face of the importunate and tactless prior. The Duke laughed the whole matter off and Leonardo returned working at his own leisure.

Others were less forgiving of his behaviour. Pope Leone X employed Leonardo in 1514 but frustration took hold of the Pope’s heart when he noticed Leonardo’s inability to attend to his duties. In desperation, Leone X exclaimed: ‘Alas! this man will never do anything, for he begins by thinking of the end of the work, before the beginning’ (Vasari, 1996). Leonardo’s presence in the Vatican lasted less than 3 years. Unlike Michelangelo and Raphael, he left no trace of his passage in Rome. Aged 64 and with nowhere to go, Leonardo must have been relieved to receive an offer from the King of France. He took with him all his drawings and one unfinished painting, *Mona Lisa* (Fig. 1), which he continued tweaking until death finally parted the master from his masterpiece.

## Lack of discipline, artistic temperament or attention deficit disorder?

Leonardo da Vinci’s exceptional artistic skills were undisputed even by his detractors. However, it would be historically incorrect to accept the biographical account elaborated by the Romantic authors of Leonardo as a solitary genius who remained unappreciated by his contemporaries owing to his ideas being too advanced for his time. His most attentive biographers had always indicated that Leonardo tried hard to please customers that were inevitably left with the disappointment of being denied possession of a concrete expression of his talent. His contemporaries could never understand or forgive his lack of discipline, not his visionary mind. In his psychoanalytical essay on Leonardo, Freud viewed what he defined Leonardo’s ‘artistic sterility’ as an infantile sexual repression caused by ‘his illegitimate birth and the pampering of his mother’ (Freud, 1922). But modern neuropsychiatry might have a different explanation.

Could Leonardo have had attention deficit and hyperactivity disorder (ADHD)? ADHD is a highly heritable childhood behavioural disorder characterized by continuous procrastination, the inability to complete tasks, mind wandering and a restlessness of the body and mind (Demontis *et al.*, 2018). In modern times, a diagnosis of ADHD prescinds from the level of intellectual ability and is increasingly more recognized among university students and adults with successful careers (Palmini, 2008). Arguably, if positively channeled, some characteristics of ADHD can bear an advantage: mind wandering can fuel creativity and originality; restlessness can move to seeking novelty and action for change.

We suggest that historical documentation supports Leonardo’s difficulties with procrastination and time management as characteristic of ADHD, a condition that might explain aspects of his temperament and the strange form of his dissipative genius. Leonardo’s difficulties were pervasive since childhood, which is a fundamental characteristic of the condition. There is also unquestionable evidence that Leonardo was constantly on the go, keeping himself occupied with doing something but often jumping from task to task. Like many of those suffering with ADHD, he slept very little and worked continuously night and day by alternating rapid cycles of short naps and waking.

In modern neuroscience, problems with executive functions are thought to underlie procrastination and impaired concentration. Neuroimaging studies of children and adolescents with ADHD indicate differences in regions of the frontal lobe and basal ganglia responsible for executive functions and impulse control. About two-thirds of children with ADHD continue to have behavioural difficulties in adulthood, which can be ameliorated with therapy (Palmini, 2008). There is enough indirect evidence to argue that Leonardo’s brain and cognitive functions were organized differently compared to the majority of the population. He was left-handed and aged 65 he suffered a severe left hemisphere stroke, which left his language abilities intact. These clinical observations strongly indicate a reverse right-hemisphere dominance for language in Leonardo’s brain, which is found in <5% of the general population. Furthermore, his notebooks show mirror writing and spelling errors that have been considered suggestive of dyslexia. Atypical hemispheric dominance, left-handedness and dyslexia are more prevalent in children with neurodevelopmental conditions, including ADHD.

And what is the possible link between left-handedness, dyslexia, ADHD and artistic abilities? Some epidemiological studies indicate that left-handed students are more likely to major in music and visual arts, while dyslexics often have superior performances in tasks for visuospatial discrimination and visual memory (Swanson, 1984). Furthermore, not only is dyslexia more prevalent among art students than students in other areas, but art students with dyslexia have superior mental imagery and 3D mental visualization of objects than art students without dyslexia (Winner and Casey, 1993). Abilities in 3D mental rotation are an important ability in those with pareidolia, an ability to recognize figures in the surrounding environment, a method that Leonardo used to boost his visual inspiration—he would contemplate for hours the changing shape of the clouds. In the initial stages of the creative process, people with ADHD may be facilitated by mind wandering and impulsivity. However, the same traits can hinder progression once the novelty of the project wanes and the interest shifts to something else. Most adults with ADHD are negatively affected by their symptoms, even if endowed with great talent.

A recent large meta-analysis shows that ADHD has a strong hereditary basis (Demontis *et al.*, 2018). The finding of the same genetic association in those who in the general population show ADHD traits and risk-taking behaviour without a diagnosis suggests ADHD sits at the extreme end of a continuum of symptoms. Within this continuum the line between those with and without a clinical diagnosis is often marked according to the impact of symptoms on the quality of life and mental wellbeing of those affected. The lack of objective biological indicators of ADHD often makes drawing that line very difficult as its negative impact depends also on a number of personal, family, professional and social circumstances, which often have a protective or a detrimental effect. There is evidence that Leonardo was often short of money and paid much less than other artists of his calibre. His behaviour negatively affected his career and relationships to the point that it is difficult to find among his contemporaries someone who had not commented on his unreliability. He was often employed in modest roles, such as the party organizer, and many of his architectural and engineering ideas were disregarded for being too unrealistic and impractical.

Undeniably Leonardo accomplished more than any other human being could possibly dream of in a lifespan, but one wonders what would have been the impact of his work on history if he had managed to apply himself more consistently to his art and effectively disseminate his intuitions and discoveries.

Besides the beauty of his art and the mesmerizing power of his observations, in the 500th anniversary of his death, Leonardo da Vinci should also be remembered for his resilience. The difficulties linked to his extraordinary wandering mind caused him deep regrets but did not prevent him from learning and exploring the wonders of human life and nature.
